# Reward system neurodynamics during menstrual pain modulated by *COMT* Val158Met polymorphisms

**DOI:** 10.3389/fnmol.2024.1457602

**Published:** 2024-09-03

**Authors:** Pei-Shan Hsu, Ching-Hsiung Liu, Ching-Ju Yang, Lin-Chien Lee, Wei-Chi Li, Hsiang-Tai Chao, Ming-Wei Lin, Li-Fen Chen, Jen-Chuen Hsieh

**Affiliations:** ^1^Institute of Brain Science, College of Medicine, National Yang Ming Chiao Tung University, Taipei, Taiwan; ^2^Integrated Brain Research Unit, Division of Clinical Research, Department of Medical Research, Taipei Veterans General Hospital, Taipei, Taiwan; ^3^Department of Chinese Medicine, Taipei Tzu Chi Hospital, Buddhist Tzu Chi Medical Foundation, New Taipei City, Taiwan; ^4^Department of Neurology, Lotung Poh-Ai Hospital, Yilan, Taiwan; ^5^Department of Biological Science and Technology, College of Engineering Bioscience, National Yang Ming Chiao Tung University, Hsinchu, Taiwan; ^6^Center for Intelligent Drug Systems and Smart Bio-devices, National Yang Ming Chiao Tung University, Hsinchu, Taiwan; ^7^Department of Physical Medicine and Rehabilitation, Cheng Hsin General Hospital, Taipei, Taiwan; ^8^Department of Obstetrics and Gynecology, Taipei Veterans General Hospital, Taipei, Taiwan; ^9^Institute of Public Health, National Yang Ming Chiao Tung University, Taipei, Taiwan; ^10^Brain Research Center, National Yang Ming Chiao Tung University, Taipei, Taiwan; ^11^Institute of Biomedical Informatics, College of Medicine, National Yang Ming Chiao Tung University, Taipei, Taiwan

**Keywords:** primary dysmenorrhea, *COMT* Val158Met polymorphism, functional magnetic resonance imaging, amplitude of low-frequency fluctuation, functional connectivity, reward system

## Abstract

**Introduction:**

Primary dysmenorrhea (PDM), characterized by cyclic pain, may involve pain modulation within the reward system (RS). The Catechol-O-methyltransferase (*COMT*) Val158Met polymorphism, which significantly influences dopamine activity, is linked to the regulation of both acute and chronic pain. This study examines the differential neurodynamic modulation in the RS associated with *COMT* Val158Met polymorphisms during menstrual pain among PDM subjects.

**Method:**

Ninety-one PDM subjects underwent resting-state fMRI during menstruation and were genotyped for *COMT* Val158Met polymorphisms. The amplitude of low-frequency fluctuation (ALFF) and functional connectivity (FC) analyses were used to assess the RS response. Psychological evaluations included the McGill Pain Questionnaire, Pain Catastrophizing Scale, Beck Anxiety Inventory, and Beck Depression Inventory.

**Result:**

Val/Val homozygotes (*n* = 50) and Met carriers (*n* = 41) showed no significant differences in McGill Pain Questionnaire, Beck Anxiety Inventory, and Beck Depression Inventory. However, Met carriers exhibited lower scores on the Pain Catastrophizing Scale. Distinct FC patterns was observed between Val/Val homozygotes and Met carriers, specifically between the nucleus accumbens (NAc) and prefrontal cortex, NAc and inferior parietal lobe, ventral tegmental area (VTA) and prefrontal cortex, VTA and precentral gyrus, and VTA and superior parietal lobe. Only Met carriers showed significant correlations between ALFF and FC values of the NAc and VTA with pain-related metrics (McGill Pain Questionnaire and Pain Catastrophizing Scale scores). NAc ALFF and NAc-prefrontal cortex FC values positively correlated with pain-related metrics, while VTA ALFF and VTA-prefrontal cortex and VTA-superior parietal lobe FC values negatively correlated with pain-related metrics.

**Discussion:**

This study reveals that the *COMT* Val158Met polymorphism results in genotype-specific functional changes in the brain’s RS during menstrual pain. In Met carriers, engagement of these regions is potentially linked to motivational reward-seeking and top-down modulation. This polymorphism likely influences the RS’s responses, significantly contributing to individual differences in pain regulation.

## 1 Introduction

Primary dysmenorrhea (PDM) is a common gynecological pain issue among young women, causing lower abdominal pain for 1–2 days each month (Itani et al., 2022). Its main mechanism may involve myometrial hypercontractility and vasoconstriction contributed by increasing inflammatory factors such as prostaglandins, cytokines, and vasopressin, but there are no observable pelvic abnormalities (Berkley, 2013). Current neuroimaging studies have found adaptive or maladaptive functional or structural changes in the brain’s pain processing network associated with this cyclical menstrual pain (Tu et al., 2010; Tu et al., 2013; Wei et al., 2016a; Lee et al., 2023), which is related to individual differences in gene polymorphism such as *BDNF* and *OPRM1* (Wei et al., 2016b; Wei et al., 2017; Hsu et al., 2023), suggesting individual differences in pain modulation may linked to genetics.

The mesolimbic dopamine pathway, often referred to as the reward pathway, links the ventral tegmental area (VTA) and nucleus accumbens (NAc) while interacting with the prefrontal cortex (PFC) (Goeders and Smith, 1983), together constituting part of the overall reward system (RS). The RS plays a crucial role in the sensory, affective-motivational, and cognitive aspects of both acute and chronic pain (Elman and Borsook, 2016; Serafini et al., 2020). Acute pain stimuli can activate the RS to modulate pain in a multidimensional manner (Becerra et al., 2001), whereas chronic pain can disrupt the brain’s RS, impairing the ability to cope with pain (Baliki et al., 2012). Studies on PDM have also noted changes in the RS, featuring both adaptive and maladaptive alterations (Zhang et al., 2019; Liu et al., 2023b).

Catechol-O-methyltransferase (COMT), a key enzyme in dopamine catabolism, is predominantly found in brain regions associated with the RS, such as the frontal cortex, striatum, amygdala, and midbrain, with particularly high levels in the frontal cortex (Matsumoto et al., 2003). In animal studies, COMT deficiency has been shown to significantly increase dopamine concentration (Gogos et al., 1998; Käenmäki et al., 2010). A common functional polymorphism in the *COMT* gene results in the substitution of valine (Val) with methionine (Met) at codon 158 (Val158Met). The Met allele homozygosity leads to a three- to four-fold reduction in COMT enzyme activity compared to Val homozygotes (Weinshilboum et al., 1999). The Met allele, associated with reduced enzyme activity, results in elevated tonic dopamine levels and decreased phasic dopamine release. This adjustment stabilizes networks involved in sustained cognitive functions, such as working memory, but may reduce the flexibility to adapt active networks (Bilder et al., 2004).

The *COMT* Val158Met polymorphism influences both acute and chronic pain modulation by altering dopamine levels in pain-related regions (Andersen and Skorpen, 2009). In acute pain scenarios, individuals who are Met homozygotes often exhibit heightened responses to experimental pain, increased mu-opioid receptor binding, but a diminished mu-opioid system response (Zubieta et al., 2003). Additionally, due to their increased pain sensitivity, the Met homozygotes often show greater reward responsiveness and engage more in reward-seeking behavior during decision-making tasks (Lancaster et al., 2012). In chronic pain conditions, the Met allele is frequently associated with increased pain sensitization and enhanced opioid efficacy (Rakvåg et al., 2008; Cohen et al., 2009; Gerra et al., 2022). The heightened pain sensitivity in Met allele homozygotes with chronic pain is thought to be related to compromised D2 receptor-mediated descending pain inhibition (Andersen and Skorpen, 2009). However, the impact of this polymorphism varies, showing less consistent results across various chronic pain syndromes (Ding et al., 2010; Patanwala et al., 2017).

This article investigates the impact of the *COMT* Val158Met polymorphism on RS neurodynamics using resting-state functional magnetic resonance imaging (rs-fMRI) and its association with menstrual pain in young females with PDM. The study focuses on key RS regions, specifically the NAc and the VTA. To measure spontaneous brain activity, an amplitude of low-frequency fluctuations (ALFF) analysis is conducted. This analysis assesses the variance of low-frequency BOLD signal fluctuations, with higher values indicating increased spontaneous brain activity (Zang et al., 2007). Additionally, functional connectivity (FC) analysis is performed using the NAc and VTA as seed regions to observe their interactions with other brain regions.

## 2 Materials and methods

### 2.1 Baseline information

#### 2.1.1 Subjects

PDM participants were included based on the following criteria: (1) having a regular menstrual cycle of approximately 27–32 days, (2) being right-handedness as determined by the Edinburgh Handedness Inventory, and (3) having a history of menstrual pain lasting more than 6 months, with an average pain score greater than 4 on a 0–10 verbal numerical scale (VNS) for the past 6 months under routine management for those with PDM. Exclusion criteria included: (1) use of any medications, contraceptives, or hormone supplements in the 6 months prior to the study, (2) pituitary gland disease, (3) organic pelvic disease, (4) psychiatric or neurological disorders, (5) head injury with loss of consciousness, (6) pregnancy or plans to conceive, (7) history of childbirth, and (8) presence of metal implants, pacemakers, claustrophobia, or any contraindications to MRI. Participants were prohibited from taking analgesics 24 h before the experiment. All PDM subjects underwent diagnosis by a gynecologist and pelvic ultrasound to rule out organic pelvic diseases.

**Figure 1:**
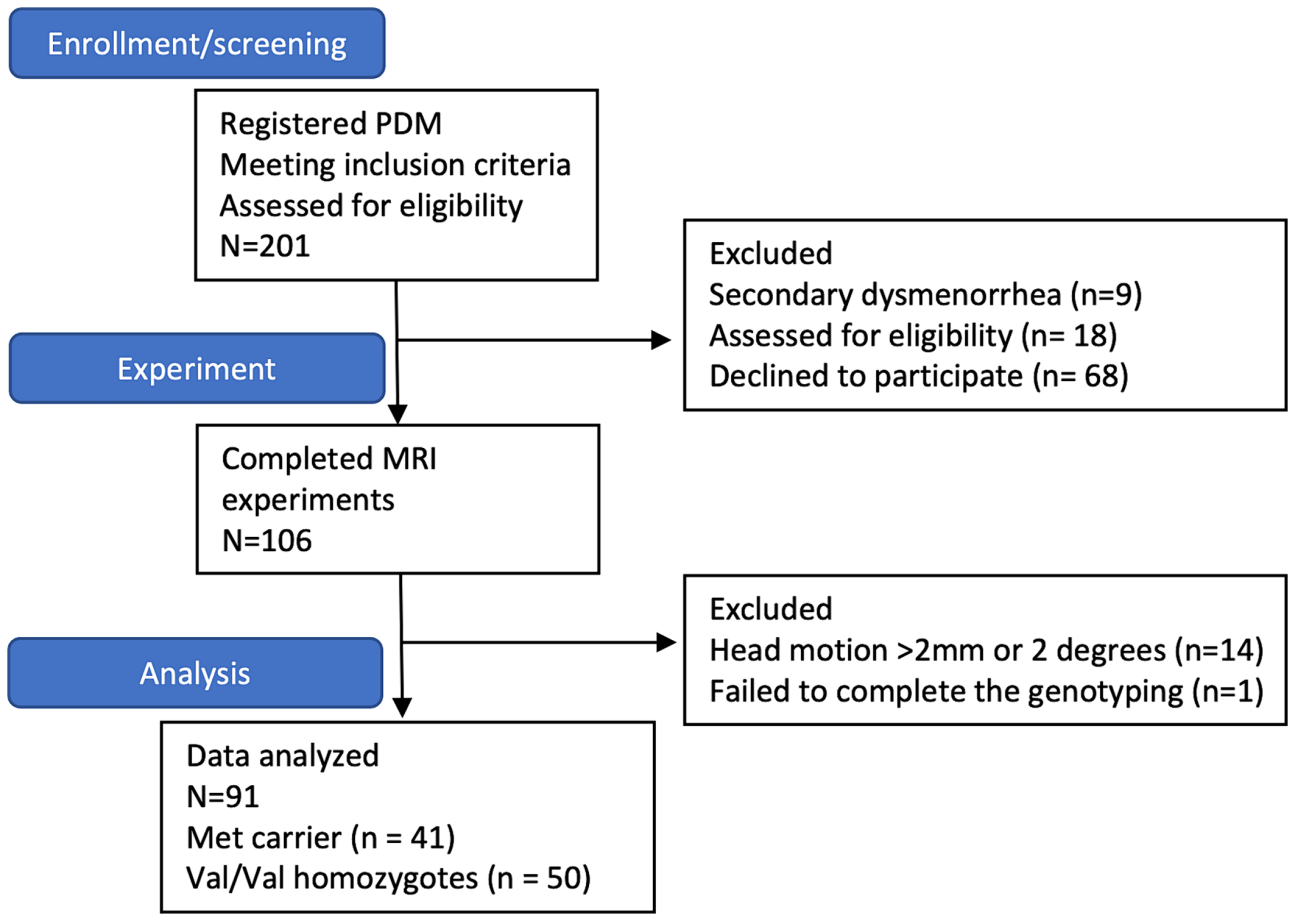
The subject flow chart. The flowchart indicates that 201 subjects with primary dysmenorrhea are initially enrolled. However, nine are excluded due to secondary dysmenorrhea; 18 are excluded due to abnormal brain findings, and eight decline participation. Ultimately, 106 subjects complete the MRI study. Among them, 14 subjects are excluded due to excessive head motion (>2 mm or 2 degrees) during scanning, and one does not successfully complete genotyping. Consequently, the final sample size comprises 91 subjects (41 Met carriers and 50 Val/Val homozygotes).

Participants with PDM were recruited through internet advertisements, initially enrolling 201 subjects. Nine were excluded due to secondary dysmenorrhea identified by pelvic ultrasound, 18 were excluded for incidental brain anomalies or abnormalities found on MRI scans, and 68 declined to participate. The final sample comprised 106 PDM subjects who completed both behavioral assessments and neuroimaging scans. Among these, 15 were further excluded due to failed genotyping or excessive head motion (>2 mm or 2 degrees) during scans. Ultimately, the study included 91 PDM patients, consisting of 4 with the Met/Met genotype, 37 with the Val/Met genotype, and 50 with the Val/Val genotype (Figure 1).

#### 2.1.2 Experimental design

MRI scans comprising T1-weighted structural images and T2-weighted gradient echo planar images (EPI) were conducted within the first 1–3 days of menstruation. The short-form McGill Pain Questionnaire (MPQ) (Melzack, 1987), the Pain Catastrophizing Scale (PCS) (Sullivan et al., 1995), Beck Anxiety Inventory (BAI) (Beck and Steer, 1993) and Beck Depression Inventory (BDI) (Beck et al., 1996) were employed to assess participants’ pain experience and psychological status during this menstrual phase. Blood samples were collected for genotyping before gonadal hormone assay. Detailed genotyping and hormone measurement methods were provided in the Supplementary material.

#### 2.1.3 Demographic and psychological measurement

Data analysis was performed using GraphPad Prism 9 (version 9.1.1). The Hardy–Weinberg equilibrium of the *COMT* Val158Met genotype distribution was assessed using the chi-square test. Demographic information, pain-related metrics, and psychological measurements of Met carrier and Val/Val homozygotes in *COMT* Val158Met polymorphism were compared. The normalcy of data distribution was assessed using the Anderson-Darling normality test. For normal distribution data, a two-sample t-test was employed. Alternatively, for non-normally distributed data, the Mann–Whitney U test was used. Results were reported as mean ± standard deviation (SD), with statistical significance defined as *p* < 0.05.

### 2.2 Image analysis

#### 2.2.1 Image acquisition

rsfMRI images were acquired using a 3.0 T MRI scanner (Magnetom Trio Tim, Siemens, Erlangen, Germany) at the National Yang Ming Chiao Tung University, with 12-channel head coil. T2*-weighted gradient echo planar imaging (EPI) sequences were used, with parameters: repetition time (TR) = 2,500 ms, echo time (TE) = 30 ms, flip angle = 90°, field of view (FOV) = 220 mm × 220 mm, matrix size = 64 × 64, slice thickness = 3.4 mm, and 200 volumes per run. High-resolution T1-weighted structural images were obtained using the magnetization-prepared rapid gradient-echo (MPRAGE) sequence (TR/TE = 2,530 ms/3.03 ms, flip angle = 7°, FOV = 224 mm × 256 mm, matrix size = 224 × 256, slice thickness = 1 mm). The initial 3 EPI scans were discarded for signal stabilization. Participants were instructed to stay awake with eyes open and heads still using head cushions and earplugs to minimize movement and noise.

#### 2.2.2 Image preprocessing

The fMRI were preprocessed using (DPARSF) V5.2 advanced edition, built on the Data Processing and Analysis of Brain Imaging (DPABI) toolbox V6.0 (Yan et al., 2016) and Statistical Parametrical Mapping 12 (SPM12^1^) in MATLAB R2018b (The MathWorks, Inc., Natick, MA, USA). The preprocessing steps included: (1) slice timing correction, (2) realignment for head motion correction, excluding participants with head motion >2 mm displacement or > 2° rotation, (3) co-registration of T1-weighted images with mean functional image, (4) segmentation into gray matter, white matter, and cerebrospinal fluid according, (5) nuisance regression was performed using the Friston 24-parameter model (Friston et al., 1996) and SPM default masks to remove head motion parameters, as well as signals from white matter and cerebrospinal fluid. (6) spatial normalization to study-specific Diffeomorphic Anatomical Registration Through Exponentiated Lie Algebra (DARTEL) template (Ashburner, 2007) transformed to the Montreal Neurological Institute (MNI-152) space, with 3 mm isotropic voxels, (7) temporal band-pass filtering (0.01 to 0.1 Hz) and (8) smoothed with a 6 mm full-width at half-maximum (FWHM). Global signal regression (GSR) was not performed to avoid exaggerating negative correlation (Murphy et al., 2009) and distorting between-group differences (Saad et al., 2012).

#### 2.2.3 Definition of NAc and VTA (seed region of interest)

Given the pivotal roles of the NAc and the VTA in pain modulation and reward processing within the brain’s RS (DosSantos et al., 2017), we created masks for these regions to conduct region of interest (ROI)-based analyses. The NAc mask was derived from the Harvard-Oxford Subcortical Atlas (Frazier et al., 2005), while the VTA mask was delineated based on the 7 T MRI atlas (Trutti et al., 2021), using manually delineation in FSLeyes^2^ (Mowinckel and Vidal-Piñeiro, 2020; Supplementary Figure 1).

#### 2.2.4 ROI-based ALFF analysis of NAc and VTA

For ALFF analysis, each voxel’s power spectrum within 0.01–0.1 Hz was calculated using a Fast Fourier Transform (FFT) (Zuo et al., 2010) and normalized to z-score maps by dividing the voxel’s power by the global mean. Averaged ALFF *z*-values were extracted from the NAc and VTA ROIs using the DPABI ROI signal extractor for between-group contrasts, with statistical significance set at *p* < 0.05.

#### 2.2.5 ROI-seed FC analysis

The image analyses in our study were performed utilizing a pre-defined RS mask created from a probabilistic map associated with “reward” from meta-analyses accessible on Neurosyn.org (Yarkoni et al., 2011).^3^ This mask encompasses VTA, NAc, amygdala, basal ganglion, insula, hippocampus, PFC, sensory/motor cortex, and superior/inferior parietal lobe (Supplementary Figure 1).

Individual FC maps were generated by calculating Pearson’s correlation coefficients (r) between the ROI located in the NAc and VTA, and other brain regions. The reference time course was obtained by averaging the time course of all voxels within each seed ROI. Correlation coefficients between the reference time course and the time course of each voxel were then computed to generate the FC map. To normalize the distribution, Fisher’s r-to-z transformation was applied to convert *r*-values to *z*-values. Between-group differences were assessed using a two-sample t-test on the FC maps derived from NAc and VTA seed, with significance set at uncorrected voxel levels of *p* < 0.001 and *p* < 0.005, followed by the family-wise error (FWE)-corrected cluster level of *p* < 0.05 in DPABI software framework. Gonadal hormones were controlled for by regressing them out as non-interest covariates in the statistical model.

#### 2.2.6 Correlation

To understand how the brain’s RS responds to menstrual pain across various *COMT* Val158Met genotypes, we correlate pain-related metrics with ALFF *z*-values from the NAc and VTA, and *z*-value FC values from regions with significant genotype differences in NAc or VTA FC for each genotype. Specifically, we examine correlations between present pain intensity (PPI) and pain rating index (PRI) sensory scores from the MPQ to assess the pain’s impact on the RS. Additionally, we explore correlations with the PCS helplessness score due to significant genotype differences observed during the menstrual phase. Pearson correlation analysis was applied, and statistical significance was defined as *p* < 0.05. To account for multiple comparisons, a Bonferroni’s correction was applied by adjusting the *p*-value to 0.0167 (0.05 divided by 3), considering that three measures (PRI sensory, PPI, PCS helpless) were analyzed.

## 3 Results

### 3.1 Demographics and clinical characteristics

As shown in Table 1, 50 Val/Val homozygotes and 41 Met carriers were included in the final analysis. The subjects in the study had a long history of menstrual pain, lasting an average of 8.38 ± 3.04 years, with pain lasting approximately 2 days per menstrual cycle (1.83 ± 0.84). More than half of the subjects with PDM (56%) reported absenteeism from school or work due to their menstrual pain, and 54.9% required analgesics. There were no significant differences in scores on the MPQ (Met carrier = 30.00 ± 15.59, Val/Val = 31.91 ± 12.91), PPI (Met carrier = 2.65 ± 1.14, Val/Val = 2.60 ± 0.99), BAI (Met carrier = 11.3 ± 7.10, Val/Val = 12.08 ± 8.85), and BDI (Met carrier = 10.20 ± 6.77, Val/Val = 11.12 ± 7.78) among the different genotypes. However, the Met carrier exhibited lower PCS scores compared to Val/Val (Met carrier = 17.25 ± 11. 10, Val/Val = 22.28 ± 10.90, *p* = 0.02), particularly in the sub-scale of Pain helplessness (Met carrier = 7.58 ± 5.57, Val/Val = 10.34 ± 5.17, *p* = 0.01) (Table 1). There were no significant differences in demographic variables such as age (Met carrier = 23.15 ± 2.32, Val/Val = 23.04 ± 2.34), education (Met carrier = 15.94 ± 1.09, Val/Val = 16.00 ± 1.09), body mass index (BMI) (Met carrier = 21.11 ± 3.70, Val/Val = 20.70 ± 2.65), Edinburgh Handedness Inventory scores (Met carrier = 77.76 ± 21.39, Val/Val = 85.06 ± 17.31), years of menstruating (Met carrier = 11.00 ± 2.71, Val/Val = 10.90 ± 2.43), menarche age (Met carrier = 12.15 ± 1.18, Val/Val = 12.20 ± 1.18), and days of menstrual cycle (Met carrier = 29.53 ± 1.37, Val/Val = 29.11 ± 1.59) among the different genotypes (Table 1).

**Table 1:** Demographic data and baseline information.

	Met (A) carrier (*n* = 41)	Val/Val (G) (*n* = 50)	*p*-value
**Age (years)**	23.15 ± 2.32	23.04 ± 2.34	0.82
**Education (years)**	15.94 ± 1.09	16.00 ± 1.09	0.68
**BMI (kg/m2)**	21.11 ± 3.70	20.70 ± 2.65	0.79
**Edinburgh Handedness Inventory (%)**	77.76 ± 21.39	85.06 ± 17.31	0.14
**Years of menstruating (year)**	11.00 ± 2.71	10.90 ± 2.43	0.96
**Menarche age (years old)**	12.15 ± 1.18	12.20 ± 1.18	0.87
**Menstrual cycle (in days)**	29.53 ± 1.37	29.11 ± 1.59	0.05
**PCS total scores (range, 0–52)**	17.25 ± 11. 10	22.28 ± 10.90	0.02
Pain rumination (range, 0–16)	6.65 ± 3.84	8.14 ± 4.24	0.09
Pain helplessness (range, 0–24)	7.58 ± 5.57	10.34 ± 5.17	0.01
Pain magnification (range, 0–12)	3.03 ± 2.36	3.80 ± 2.42	0.10
**MPQ**			
PRI scores (range, 0–78)	30.00 ± 15.59	31.91 ± 12.91	0.56
Sensory (range, 0–42)	16.70 ± 7.95	17.62 ± 6.48	0.57
Affective (range, 0–14)	3.78 ± 3.16	3.96 ± 2.57	0.47
Evaluation (range, 0–5)	2.27 ± 1.91	2.75 ± 2.12	0.34
Miscellaneous (range, 0–17)	7.24 ± 4.73	7.58 ± 3.85	0.72
PPI scores (range, 0–5)	2.65 ± 1.14	2.60 ± 0.99	0.69
**BAI**	11.3 ± 7.10	12.08 ± 8.85	0.98
**BDI**	10.20 ± 6.77	11.12 ± 7.78	0.56

Data are presented as the mean ± SD. BMI, body mass index; PCS, pain catastrophizing scale; MPQ, McGill pain questionnaire; PRI, pain rating index; PPI, present pain intensity; BAI, Beck Anxiety Inventory; BDI, Beck Depression Inventory BDI.

### 3.2 Genetic data

The distribution of the *COMT* Val158Met gene in the PDM subjects (*p* = 0.97) was consistent with the Hardy–Weinberg equilibrium. Due to the limited number of Met/Met homozygotes, the Val/Met heterozygotes and Met/Met homozygotes were combined and analyzed together as Met allele carriers.

### 3.3 Differences in ALFF analysis in ROI

There were no significant differences in the extracted mean *z*-values of ALFF between the genotypes for either the left or right NAc and VTA (Supplementary Figure 2).

### 3.4 Differences in ROI-seed FC

**Figure 2:**
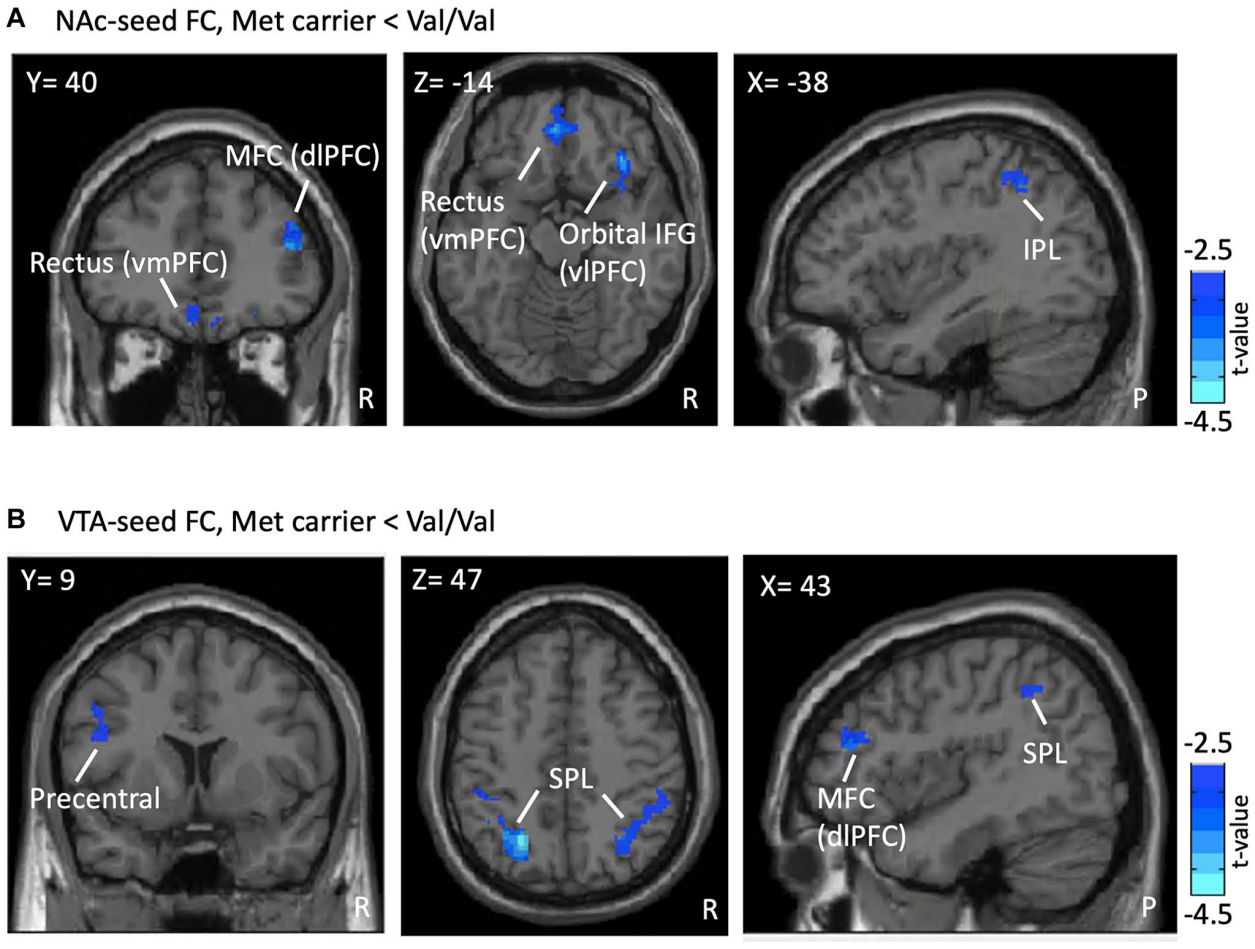
FC differences in the RS between genotypes. Presented are the outcomes of FC analyses, illustrating differences associated with the *COMT* Val158Met polymorphism within a reward-oriented mask defined on Neurosyn.org. **(A)** Shows lower NAc-seeded FC in Met carriers between the NAc and regions including the MFC (dlPFC), rectus (vmPFC), orbital IFG (vlPFC), and IPL compared to Val/Val homozygotes (blue region). **(B)** Reveals lower VTA-seeded FC in Met carriers between the VTA and the MFC (dlPFC), precentral gyrus, and SPL. Results are thresholded at the uncorrected voxel level *p* = 0.005, followed by the FWE-corrected cluster level *p* = 0.05. The color bar denotes the t-score. ROI, region of interest; FC, functional connectivity; RS, reward system; NAc, nucleus accumbens; MFC, middle frontal gyrus; dlPFC, dorsolateral prefrontal cortex; vmPFC, ventromedial prefrontal cortex; IFG, inferior frontal gyrus; vlPFC, ventrolateral prefrontal cortex; IPL, inferior parietal lobule; VTA, ventral tegmental area; SPL, superior parietal lobule; R, right; P, posterior.

In NAc-seeded FC, Met-carriers showed decreased connectivity between the NAc and the orbital part of the inferior frontal gyrus (IFG) [ventrolateral prefrontal cortex (vlPFC)], rectus [ventromedial prefrontal cortex (vmPFC)], middle frontal gyrus (MFC) [dorsolateral prefrontal cortex (dlPFC)], and inferior parietal lobule (IPL) compared to Val/Val homozygotes (Table 2 and Figure 2A). In VTA- seeded FC, Met-carriers exhibited decreased connectivity between the VTA and the MFC (dlPFC), precentral gyrus, and superior parietal lobule (SPL) compared to Val/Val homozygotes (Table 2 and Figure 2B).

**Table 2:** FC differences in the RS between genotypes.

Seed	Contrast of genotype	Region, laterality	BA	Cluster	*t* score	Peak coordinate		
						*x*	*y*	*z*
NAc	Met carrier < Val/Val	NS						
	Met carrier < Val/Val	R Orbital IFG (vlPFC)*	47	170	4.35	34	32	−18
		L Rectus (vmPFC)*	11	305	4.00	−4	46	−16
		R MFC (dlPFC)	46	182	3.73	44	40	18
		L IPL	40	85	3.27	−36	−48	46
VTA	Met carrier > Val/Val	NS						
	Met carrier < Val/Val	R MFC (dlPFC)*	46	158	3.98	48	40	16
		L Precentral		75	3.02	−48	2	28
		R SPL		359	3.93	32	−60	56
		L SPL*	7	389	4.34	−26	−60	48

Peak coordinates refer to the MNI space. Significance was set at the uncorrected voxel level *p* < 0.005 followed by the family-wise error corrected cluster level *p* < 0.05; FC, functional connectivity; RS, reward system; NAc, nucleus accumbens; VTA, ventral tegmental area; BA, Brodmann area; NS, non-significant; R, right; L, left; IFG, inferior frontal gyrus; vlPFC, ventrolateral prefrontal cortex; vmPFC, ventromedial prefrontal cortex; MFC, middle frontal gyrus; dlPFC, dorsolateral prefrontal cortex; IPL, inferior parietal lobule; SPL, superior parietal lobule. *Denotes significant results thresholded at an uncorrected voxel threshold of *p* < 0.001.

### 3.5 Correlation

**Figure 3:**
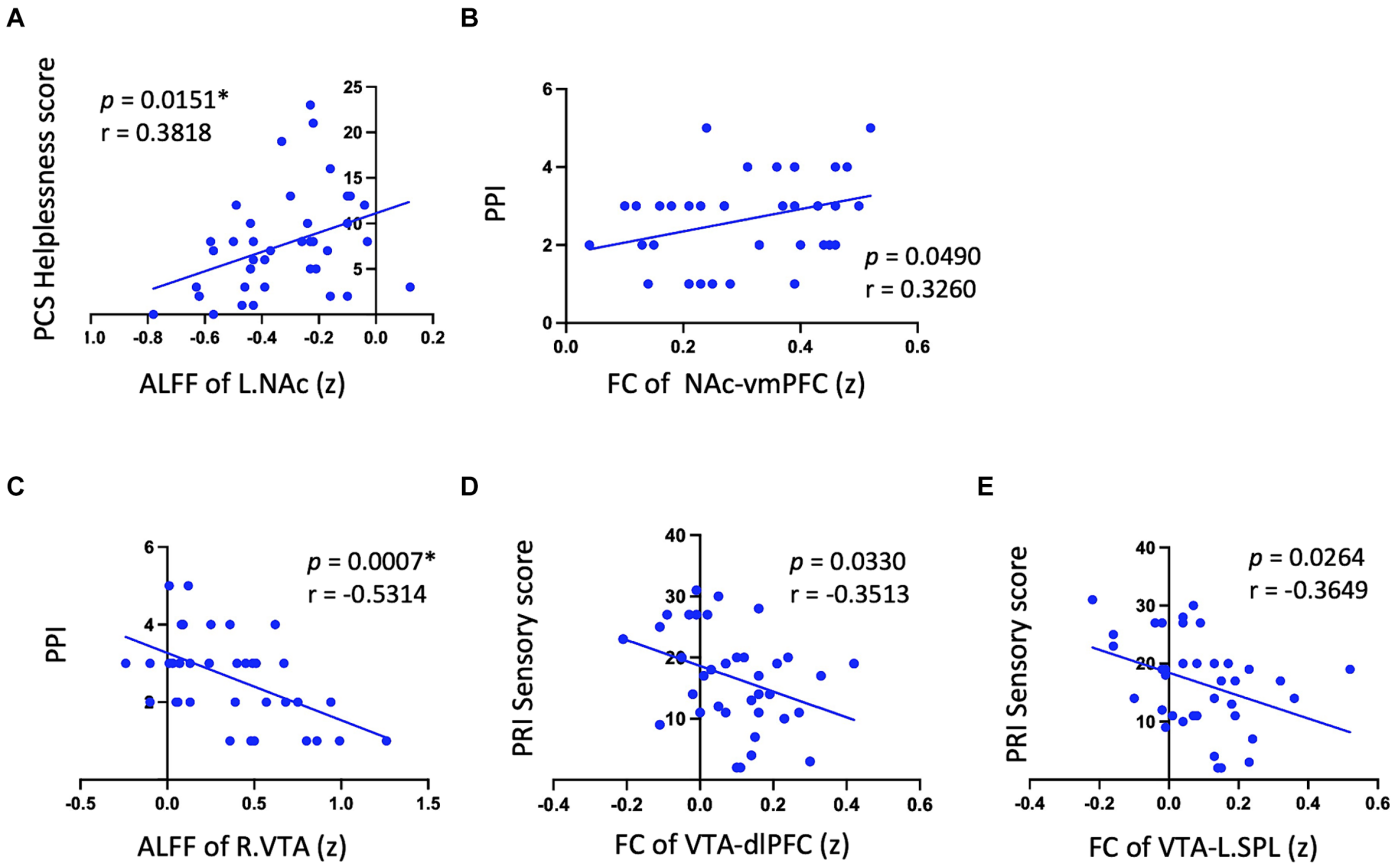
Significant correlation between psychological pain metrics and RS neurodynamic metrics in met carriers. Significant results are observed only in Met carriers. **(A)** Shows a positive correlation between NAc ALFF values and PCS helplessness scores. **(B)** Indicates that NAc FC to the Rectus (vmPFC) is positively correlated with PPI. **(C)** Displays a negative correlation between VTA ALFF values and PPI. **(D,E)** Reveal that VTA FC to the medial frontal cortex (dlPFC) and bilateral SPL are negatively correlated with PRI sensory scores. This figure displays only a significant subset of the correlation data to illustrate the positive and negative correlation trends between neurodynamic metrics and pain metrics in the NAc and VTA. Detailed data are provided in Table 3. RS, reward system; L, left; NAc, nucleus accumbens; ALFF, amplitude of low-frequency fluctuations; PCS, pain catastrophizing scale; FC, functional connectivity; vmPFC, ventromedial prefrontal cortex; PPI, present pain intensity; R, right; VTA, ventral tegmental area; dlPFC, dorsolateral prefrontal cortex; SPL, superior parietal lobule; PRI, pain rating index. (z) indicates z-transformed mean values for ALFF and FC. *Signifies significant results after Bonferroni correction (*p* < 0.016).

In Met carriers of PDM during the menstrual phase, NAc showed a positive correlation between ALFF values and PCS helplessness scores (*p* = 0.15, *r* = 0.39) (Table 3 and Figure 3A). The VTA exhibited negative correlations between ALFF values and PRI sensory scores (left: *p* = 0.02, *r* = −0.39; right: *p* = 0.02, *r* = −0.39), PPI (left: *p* < 0.01, *r* = −0.50; right: *p* < 0.01, *r* = −0.53), and PCS helplessness scores (left: *p* = 0.01, *r* = −0.40; right: *p* = 0.02, *r* = −0.39) (Table 3 and Figure 3C). Additionally, NAc FC to the Rectus (vmPFC) was positively correlated with PPI (*p* = 0.05, *r* = 0.33) (Table 3 and Figure 3B), while VTA FC to the medial frontal cortex (dlPFC) (*p* = 0.03, *r* = −0.35) and bilateral SPL were negatively correlated with PRI sensory scores (right: *p* = 0.03, *r* = −0.35; left: *p* = 0.03, *r* = −0.36) (Table 3 and Figures 3D,E). No significant correlations were found between ALFF or FC values and pain-related metrics in Val/Val homozygotes (Table 3).

**Table 3:** Correlation between RS neurodynamic metrics and psychological pain metrics.

Genotype	Region	Method	Psychological pain metrics	*p*-value	*r*
Region of interest					
Met carrier	L. NAc	ALFF	PCS helplessness score	0.0151*	0.3818
	R. NAc	ALFF	NS		
	L. VTA	ALFF	MPQ_PRI sensory score	0.0183	−0.3858
			PPI	0.0017*	−0.4985
			PCS helplessness score	0.0103*	−0.4013
	R. VTA	ALFF	MPQ_PRI sensory score	0.0186	−0.3852
			PPI	0.0007*	−0.5314
			PCS helplessness score	0.0195	−0.3680
Val/Val	NS				
Significant regions of FC analysis					
Met carrier	L Rectus (vmPFC)	NAc FC	PPI	0.0490	0.3260
	R MFC (dlPFC)	VTA FC	MPQ_PRI_Sensory score	0.0330	−0.3513
	R SPL	VTA FC	MPQ_PRI_Sensory score	0.0345	−0.3485
	L SPL	VTA FC	MPQ_PRI_Sensory score	0.0264	−0.3649
Val/Val	NS				

RS, reward system; R, right; L, left; NAc, Nucleus accumbens; VTA, ventral tegmental area; ALFF, amplitude of low frequency fluctuations; PCS, pain catastrophizing scale; MPQ, McGill pain questionnaire; PRI, pain rating index; PPI, present pain intensity; NS, non-significant; FC, functional connectivity; vmPFC, ventromedial prefrontal cortex; MFC, middle frontal gyrus; dlPFC, dorsolateral prefrontal cortex; SPL, superior parietal lobule. *Signifies significant results after Bonferroni correction (*p* < 0.016).

## 4 Discussion

This study demonstrates that the *COMT* Val158Met polymorphism leads to genotype-specific differences in the FC (Val > Met) and distinct correlation patterns between neurodynamic metrics and pain metrics within key RS areas, specifically the NAc and VTA, during menstrual pain. Met carriers exhibit dynamic changes that correlate with pain metrics, suggesting an active role of their RS in driving motivation and top-down regulation. In contrast, the Val genotype, which shows greater individual variations and lacks a clear correlation with the pain metrics, aligning with the proposition of a higher functionality.

### 4.1 The RS psychoneurodynamics of Met carriers implicating pain-avoidance motivation

Although there were no differences in NAc ALFF values between genotypes (Supplementary Figure 2), Met carriers showed a positive correlation between NAc ALFF values and PCS helplessness scores (Table 3 and Figure 3A). Additionally, the FC between the NAc and vmPFC positively correlated with PPI (Table 3 and Figure 3B). These suggest that Met carriers may be associated with a stronger motivation to alleviate menstrual pain as a reward (Leknes et al., 2011), aligning with increased reward-seeking and more expressed pleasant responses in Met genotype individuals (Frank et al., 2009; Lancaster et al., 2012). Reward seeking and motivation are primarily driven by dopaminergic signals from the VTA to the NA (Navratilova and Porreca, 2014). The vmPFC plays a crucial role in processing rewards and regulating emotions, while dopaminergic projections from the PFC to the NAc modulate these functions (Huang et al., 2019). Given that COMT is primarily found in the PFC (Matsumoto et al., 2003), the *COMT* Val158Met polymorphism may modulate pain-relief motivation during menstrual pain.

### 4.2 Enhanced pain modulation in Met carrier

Despite no differences in VTA ALFF values between genotypes (Supplementary Figure 2), Met carriers exhibited negative correlations between VTA ALFF values and PRI sensory scores (Table 3 and Figure 3C), PCS helplessness scores, and PPI. Additionally, FC between the VTA and dlPFC/SPL negatively correlated with PRI sensory scores (Table 3 and Figures 3D,E). Higher VTA ALFF and FC values are associated with lower PCS, PRI, and PPI, suggesting that VTA activity may modulate the pain experience in Met carriers. The VTA, a dopaminergic center of the motivational circuit (Navratilova and Porreca, 2014), interacts with the SPL, which processes and integrates sensory information (Teixeira et al., 2014; Passarelli et al., 2021). The VTA also interacts with the dlPFC, which is involved in emotional regulation (Buhle et al., 2014; Frank et al., 2014) and top-down pain suppression (Lorenz et al., 2003; Seminowicz and Moayedi, 2017).

The VTA interacts with the NAc (Navratilova and Porreca, 2014) and possibly projects to the periaqueductal gray (PAG) (Omelchenko and Sesack, 2010; Ntamati et al., 2018; Cappon et al., 2019), a core region of the descending pain modulation and opioid modulation systems (Linnman et al., 2012). The negative correlations between VTA neurodynamic metrics and pain psychological metrics in Met carriers may be mediated by the interaction between the dopamine system and the mu-opioid system (Zubieta et al., 2003; Rakvåg et al., 2008). This is supported by findings that the Met allele, associated with lower COMT enzyme activity, shows higher mu-opioid receptor binding in striatal regions, which potentially regulates the motivational circuit by integrating sensory information and motor response with affective and cognitive influences (Zubieta et al., 2003).

While both the VTA and NAc are involved in motivation and reward (Navratilova and Porreca, 2014), they may have different modulation mechanisms through their synergy with various brain areas (Haber and Knutson, 2010; Mitsi and Zachariou, 2016). The differing responses of the VTA and NAc to menstrual pain may reflect the RS’s multi-dimensional adaptation in PDM subjects. However, the stronger coupling between neurodynamic expressions and psychological manifestations in Met carriers may implicate reduced flexibility in their RS. The Met allele of the *COMT* gene is associated with lower COMT activity, which is hypothesized to increase tonic dopamine levels while decreasing phasic dopamine release. This alteration enhances brain network stability but reduces flexibility (Bilder et al., 2004). Such a reduction in flexibility may increase the risk of developing chronic pain (Bilder et al., 2004; Andersen and Skorpen, 2009). Given that functional pain disorders and chronic pain conditions such as irritable bowel syndrome, fibromyalgia, chronic fatigue syndrome, and lower back pain may co-occur with PDM later in life (Altman et al., 2006; Berkley, 2013; Chung et al., 2014; Tu et al., 2020), further longitudinal exploration of the potential association between Met carriers and chronic pain can be both heuristic and significant.

### 4.3 Lower helplessness score in Met carriers

In our study, there were no significant differences in MPQ and PPI scores between genotypes (Table 1), suggesting that the *COMT* Val158Met polymorphism does not impact pain intensity in young PDM subjects. However, Met carriers had significantly lower total PCS scores, particularly in the helplessness domain (Table 1). The PCS assesses cognitive-affective responses to pain, emphasizing negative aspects such as helplessness (Sullivan et al., 1995). These responses may be underpinned by the brain’s attention network (Rischer et al., 2020) and limbic system (Bushnell et al., 2013; Galambos et al., 2019), which can interfere with the descending pain inhibitory system (Toledo et al., 2020). An active RS can enhance positive affect and motivation (Navratilova and Porreca, 2014), potentially counteracting negative emotional states like helplessness (Kuhl, 1981; Butler and Roediger, 2008). Therefore, the lower PCS helplessness scores in Met carriers may be ascribed to stronger motivation and enhanced pain modulation.

### 4.4 Val homozygotes with diverse responses

Val homozygotes exhibit higher NAc/VTA-seed FC without a clear correlation to pain metrics, potentially indicating diversity in brain-behavior relationship. The enhanced FC regions in Val homozygotes are related to pain modulation, such as the SPL and IPL for sensory processing (Teixeira et al., 2014; Berlucchi and Vallar, 2018), dlPFC and mPFC for emotional and cognitive top-down pain modulation (Lorenz et al., 2003; Ong et al., 2019), and the precentral gyrus for descending pain modulation in PDM subjects (Wei et al., 2016a; Hsu et al., 2023; Figures 2A,B). Val allele carriers may engage dlPFC and insula as compared to Met allele carriers during pain (Schmahl et al., 2012). Additionally, a previous study reported that the rs4680 Val allele, when combined with different rs4818 and rs6269 sequences, leads to varying levels of pain sensitivity (Diatchenko et al., 2005). This variability in sensitivities may account for the lack of a direct correlation between RS neurodynamics and psychological pain metrics. Thus, Val homozygotes demonstrate greater flexibility, potentially adaptive, in the RS, consistent with the tonic-phasic dopamine hypothesis (Bilder et al., 2004).

### 4.5 Limitations and further consideration

This study has several limitations. First, due to the lower prevalence of Met/Met homozygotes in East Asians for the *COMT* Val158Met polymorphism (Wang et al., 2016), our study lacked a sufficient number of Met/Met individuals, requiring us to group genotypes into Met carriers and Val groups. The predominance of Val/Met genotypes among Met carriers suggests that their RS regulation may primarily reflect Val/Met characteristics. Second, using pain metrics to infer the RS’s motivational response requires further behavioral testing to confirm elevated motivation (Leknes et al., 2011). Third, considering that PDM is not classified as a neuropsychiatric disease with prominent functional and structural changes, we aimed to capture subtle yet potentially significant information to provide valuable insight for future research (Marek et al., 2022). Therefore, we presented two levels of statistical information in the results: a relatively permissive uncorrected voxel level of *p* < 0.005 and more stringent uncorrected voxel level of *p* < 0.001, both followed by a family-wise error (FWE) corrected cluster level of *p* < 0.05. This approach has been adopted by many authors in the field of neuroimaging (e.g., Roiser et al., 2016; Wei et al., 2016b; Liu et al., 2023a). However, the risk of false positives should be carefully considered in data interpretation (Marek et al., 2022). Fourth, considering that *COMT* polymorphisms’ influence on pain sensitivity may extend beyond single SNP variations and affect opioid consumption (Zubieta et al., 2003; Diatchenko et al., 2005), future studies should analyze additional haplotype sequences and explore gene–gene interactions within *COMT*.

## 5 Conclusion

This study provides novel insight into the neurodynamic impact of the *COMT* Val158Met polymorphism on VTA and NAc-based RS’s FC. During the menstrual cycle, the functional dynamics of the RS exhibit varying modulation flexibility associated with the individual’s *COMT* Val158Met genotype. These genetic factors influencing brain resilience may contribute to individual pain experience and coping mechanisms in PDM, potentially affecting future vulnerability to other chronic pain disorders. Further research is required to determine if the *COMT*-genotype-specific modulation of RSs correlates with other chronic pain conditions later in life.

## Data Availability

The raw data supporting the conclusions of this article will be made available by the authors, without undue reservation.

## Footnotes

^1^ http://www.fil.ion.ucl.ac.uk/spm

^2^ https://git.fmrib.ox.ac.uk/fsl/fsleyes/fsleyes/

^3^ https://neurosynth.org
