# Supplementary material for: Reward system neurodynamics during menstrual pain modulated by COMT Val158Met polymorphisms
Source: Front Mol Neurosci. 2024 Sep 3;17:1457602. doi: 10.3389/fnmol.2024.1457602 (PMC11405383; doi:10.3389/fnmol.2024.1457602)
Supplement: Supplementary file 1 [file Data_Sheet_1.PDF]

**Supplementary material**

## Genotyping

Whole blood was collected in 4 mL EDTA tubes and stored at 4 °C in a refrigerator. Subsequent DNA extraction was carried out using the Puregene kit following the manufacturer's guidelines (Gentra Systems, Minneapolis, MN). Commercial TaqMan single-nucleotide polymorphism assays (Applied Biosystems, Foster City, CA) were utilized for genotyping. Polymerase chain reaction amplification was conducted in a total volume of 10 μL using the following amplification protocol: 50 °C for 2 min, 95 °C for 10 min, and 40 cycles of 92 °C for 15 sec and 60 °C for 1 min. Fluorescence measurements were taken using the ABI HT7900 (Applied Biosystems, Foster City, CA), and allele calling was performed by the SDS 2.2 software package (Applied Biosystems). Genotypes were independently assigned to the subjects by two technicians who were blinded to the subject's personal information.

## Serum gonadal hormone measurements

The sera extracted from the blood samples collected during the menstrual phase were preserved for batch analysis using commercial assays (specifically, UniCel DxC 800 Synchron Clinical Systems by Beckman Coulter, Inc., based in Brea, CA, USA). Total serum concentrations of estradiol and progesterone were measured using chemiluminescence immunoassays, while total serum testosterone levels were determined through radioimmunoassays. Considering previous research indicating the impact of gonadal hormones on resting-state fMRI results (Veldhuijzen et al., 2013; Petersen et al., 2014), we controlled for these hormonal effects by regressing them out as non-interest covariates in our image analysis.

**Supplementary Figure 1.**

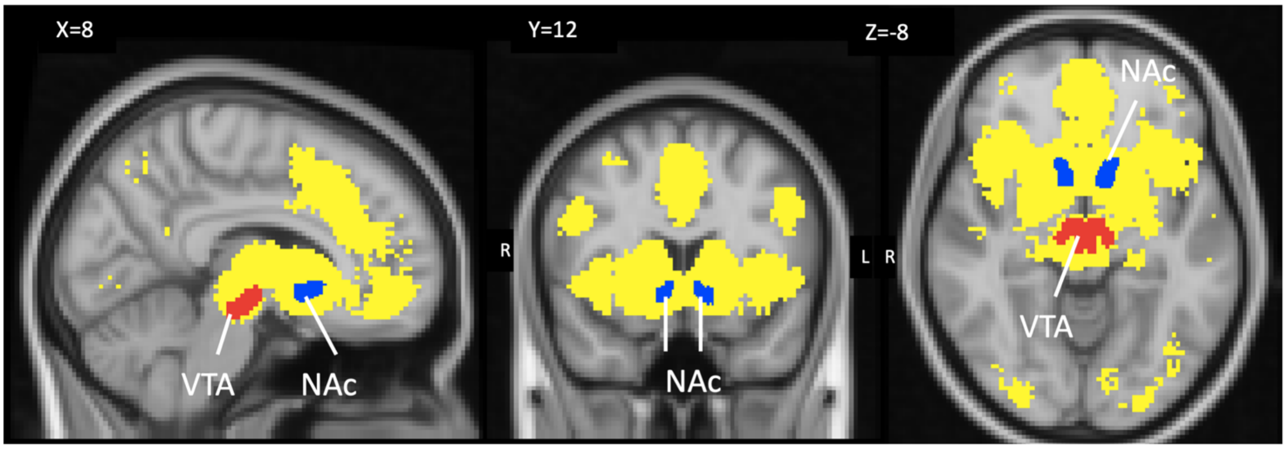


**Supplementary Figure 1. Reward Network Mask and Regions of Interest.**
This figure presents the reward network mask (yellow), covering areas involved in reward processing, such as VTA, NAc, amygdala, basal ganglia, insula, hippocampus, prefrontal cortex, sensory/motor cortex, and posterior parietal lobe. This mask is based on probabilistic maps from prior meta-analyses on Neurosynth.org. The NAc and VTA, highlighted in blue and red, respectively, were delineated using Harvard-Oxford subcortical atlas and validated against recent literature (Trutti et al., 2021). VTA, ventral tegmental area; NAc, nucleus accumbens.

**Supplementary Figure 2.**

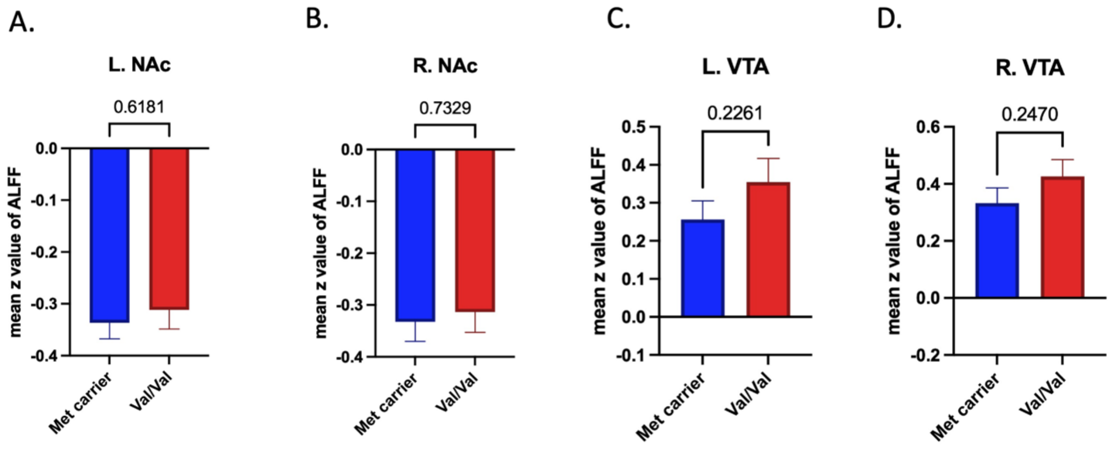


**Supplementary Figure 2. Between-genotype tests of ALFF value from NAc and VTA in individuals with primary dysmenorrhea.** The mean ALFF z-values (mean ± SEM) from the NAc and VTA (region of interest) in individuals with primary dysmenorrhea revealed no differences between carriers of the Met allele and those with the Val/Val homozygotes of *COMT* Val158Met polymorphism during the menstrual phase. ALFF, amplitude of low-frequency fluctuations; R, right; L, left; NAc, nucleus accumbens; VTA, ventral tegmental area.
